# Objectively measured peri-vaccination sleep does not predict COVID-19 breakthrough infection

**DOI:** 10.1038/s41598-024-53743-4

**Published:** 2024-02-26

**Authors:** Stuti J. Jaiswal, Matteo Gadaleta, Giorgio Quer, Jennifer M. Radin, Jill Waalen, Edward Ramos, Jay Pandit, Robert L. Owens

**Affiliations:** 1 The Scripps Research Institute, La Jolla, CA USA; 2 University of California San Diego School of Medicine, La Jolla, CA USA

**Keywords:** Public health, Outcomes research

## Abstract

Prior studies have shown that sleep duration peri-vaccination influences an individual’s antibody response. However, whether peri-vaccination sleep affects real-world vaccine effectiveness is unknown. Here, we tested whether objectively measured sleep around COVID-19 vaccination affected breakthrough infection rates. DETECT is a study of digitally recruited participants who report COVID-19-related information, including vaccination and illness data. Objective sleep data are also recorded through activity trackers. We compared the impact of sleep duration, sleep efficiency, and frequency of awakenings on reported breakthrough infection after the 2nd vaccination and 1st COVID-19 booster. Logistic regression models were created to examine if sleep metrics predicted COVID-19 breakthrough infection independent of age and gender. Self-reported breakthrough COVID-19 infection following 2nd COVID-19 vaccination and 1st booster. 256 out of 5265 individuals reported a breakthrough infection after the 2nd vaccine, and 581 out of 2583 individuals reported a breakthrough after the 1st booster. There was no difference in sleep duration between those with and without breakthrough infection. Increased awakening frequency was associated with breakthrough infection after the 1st booster with 3.01 ± 0.65 awakenings/hour in the breakthrough group compared to 2.82 ± 0.65 awakenings/hour in those without breakthrough (*P* < 0.001). Cox proportional hazards modeling showed that age < 60 years (hazard ratio 2.15, *P* < 0.001) and frequency of awakenings (hazard ratio 1.17, *P* = 0.019) were associated with breakthrough infection after the 1st booster. Sleep duration was not associated with breakthrough infection after COVID vaccination. While increased awakening frequency during sleep was associated with breakthrough infection beyond traditional risk factors, the clinical implications of this finding are unclear.

## Introduction

Inadequate sleep may have a negative impact on the development of immunity to vaccination^1^. For example, restricting sleep to four hours per day prior to influenza vaccination reduced subsequently measured IgG antibody titers by more than half compared to individuals with normal sleep times^2^. In another study of hepatitis B vaccination, short sleep duration as measured by actigraphy was associated with decreased antibody production after vaccination as well as decreased circulating antibody levels at six months post-vaccination^3^. Recently, Benedict and Cedernaes re-visited the idea of sleep duration and vaccines in the context of immunization against SARS-CoV-2^4^, with a call for more data in this area as there is a paucity of information in regards to the impact of sleep surrounding vaccination on subsequent vaccine effectiveness. However, to our knowledge, there has been no large-scale, real-world analysis of sleep and the clinically relevant outcome of breakthrough infection (rather than antibody levels, of which data are mixed in regards to predicting infection^5–8^).

Our group developed the DETECT study at the onset of the COVID-19 pandemic to track biometric data from wearable activity trackers along with self-reported symptoms, test results, and vaccination information^9^. More than 40,000 individuals have enrolled between March 2020 and October 2022. Sleep biometrics included sleep duration, sleep efficiency, and frequency of awakenings per hour in a sleep period. Here, we use the DETECT cohort to address the primary hypothesis that short sleep duration, as measured by wearable activity trackers, may predict risk of breakthrough infection after full vaccination against SARS-CoV-2.

## Methods

The DETECT study was approved by the Scripps Institutional Review Board (IRB) on March 2020 (IRB 20–7531), and methods have been published previously^9^. All research was performed in accordance with IRB regulations, and in accordance with the Declaration of Helsinki. Informed consent was obtained from all participants. Briefly, eligible participants are 18 years of age or older and currently live in the United States. Individuals can participate in the study by downloading the MyDataHelps application (app) on either an iOS or Android smartphone. Informed consent is obtained digitally through the application. In addition to basic demographic information, participants are asked to report symptoms, testing results, vaccination information, and are asked to connect their personal activity trackers to the app to share their data. Currently Fitbit, and devices using the Apple HealthKit or Google Fit are compatible for data sharing in the app. Participants consent to receive push notifications from the study, and they also receive a newsletter regarding important updates and information on COVID-19.

### Collection of vaccination information

The DETECT application intermittently sends push notifications to the participant’s smartphone to input their vaccination information. They are asked to input the date they received a vaccination against SARS-CoV-2, whether it is the 1st, 2nd, or booster dose, and to identify which vaccine they received (i.e., Pfizer-BioNTech, Moderna, or Johnson and Johnson). We included individuals who reported an mRNA or a Johnson and Johnson vaccine date on or after December 1, 2020. The Johnson & Johnson vaccine was counted in the “2nd dose,” category as this reflects the clinical status of “fully vaccinated.”

### Collection of COVID-19 positivity and breakthrough infection data

A participant can go into the DETECT application at any time to input whether they are feeling symptoms (e.g., cough, fever, chills, rhinorrhea). If participants are symptomatic, they are asked if and when they took a COVID-19 test. Individuals who reported a COVID-19 blood test (e.g., antibody test) were excluded as this would likely not reflect acute infection. If participants took an antigen or PCR test, they are asked to report whether the test was positive, negative, or invalid. Study participants can also go into the app any time to report a negative or positive test regardless of symptom reporting. Push notifications are sent weekly to remind participants to enter any symptom or testing data that they may have. Here, we defined a breakthrough infection as a positive COVID-19 test that occurred after any vaccination, but prior to the next vaccination. For analysis purposes, the sleep metrics of the most recent vaccine dose prior to infection were considered.

### Collection of sleep data

For the purposes of consistency in the analysis, only individuals who connected a Fitbit device were included. Any Fitbit device with heart rate measuring capability were included. Prior studies have conducted comparisons between sleep measured from wearable activity trackers to both actigraphy and polysomnography^3,10–12^. In terms of accuracy in healthy populations, some studies suggest that wearables over-estimate sleep duration compared to PSG on the order of only eight minutes^10–16^. Furthermore, recent studies have shown that many newer devices performed as well as or better than actigraphy on sleep vs. wake measures in children to adults, though not quite to PSG accuracy^12,17^.

#### Defining sufficient sleep data

Similar to prior work by Prather et al. in their investigations of sleep surrounding Hepatitis B vaccination^3^, a participant was considered to have sufficient sleep data to be included in the analysis if the following two criteria were met: (1) they had at least 1 day of valid sleep data in the 3 days preceding the vaccine date, and (2) they had at least 1 day of valid sleep data in the 3 days following the vaccine date. Daily sleep data were considered valid if the device was able to recognize sleep stages (light, deep, REM stage) for at least one sleep session during the day. Scenarios that prevent the device from tracking sleep stages are the following: inconsistent or lack of heart rate reading, device worn too loosely, sleep entered manually (and not through the automatic sleep detection algorithm), a total sleep duration of less than 3 h, or if the device battery is critically low.

We extracted the following sleep metrics from individuals with sufficient sleep data:

1. *Main sleep duration* This was the longest sleep period in a day and excluded any naps.
2. *Sleep efficiency* The fraction of time in bed that is spent asleep during the main sleep period.
3. *Frequency of awakenings* Reported as the average number of awakenings per hour during the main sleep period.

### Data analysis and statistics

All analyses were conducted using Python and R. The sleep metrics described above are reported as mean and standard deviation and are graphically shown over a 30-day time course surrounding vaccination (15 days prior to vaccination and 15 days after vaccination). Similar to the prior Hepatitis B vaccination study, the peri-vaccination period was defined as the 3 days pre- and post-vaccination^3^. We used Kruskal–Wallis testing to compare sleep metrics in the peri-vaccination period between individuals who reported a breakthrough infection and those who did not report a breakthrough infection. In an effort to remain consistent with prior work, we confined our main analysis to assessing the impact of sleep during the peri-vaccination period on breakthrough infection. However, we also considered if (1) pre-vaccination sleep (using sleep in the three days prior to the vaccination), or (2) “delta (Δ sleep),” defined as the difference in sleep metrics between the three days following and the three days preceding vaccination, had an impact on reported breakthrough infection.

Next, we implemented a logistic regression model, with independent variables of age, gender and sleep metrics (defined above as total sleep duration, sleep efficiency, and frequency awakenings) and dependent variable of COVID-19 infection. This model was applied separately for each vaccine dose analyzed, and covered available sleep data 3 days pre- and post-vaccination. To evaluate the independent contribution of sleep metrics, a model with only age and gender as independent variables was developed for comparison.

Finally, we adjusted our breakthrough infection outcome for time of vaccination to time of infection using a Cox Proportional Hazards model using time from vaccination with the second dose. Further survival analyses for time to breakthrough infection were completed using Kaplan–Meier curves that were constructed based on age (age < 60 years compared to age ≥ 60 years). Shaded areas in all curves represent the 95% confidence interval evaluated with 10.000 bootstrap iterations.

## Results

### Study population

**Figure 1:**
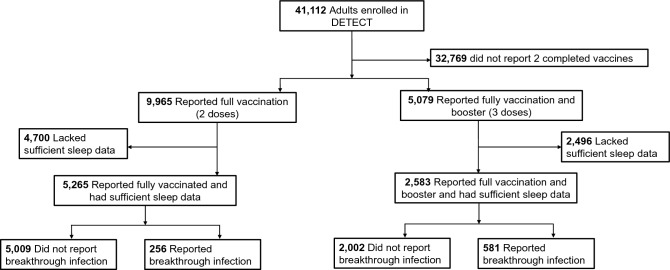
Participant flow diagram. Of the 41,112 individuals enrolled in the DETECT cohort, 233 reported breakthrough infection after the 2nd vaccine dose while 581 reported breakthrough after the 1st booster. Relatively few subjects had reported infection between 1st and 2nd vaccine (*N* = 23), or after a 2nd booster (*N* = 178)—these data are not shown.

There were 41,112 individuals enrolled in DETECT from March 2020 until October 2022. A participant flow diagram is shown in Fig. 1. There were relatively few individuals who reported a breakthrough infection between the 1st and 2nd dose of the mRNA vaccine (*N* = 23), and after the 2nd booster (*N* = 178). Here, we focused on breakthrough after full vaccination (considered the 2nd dose for the mRNA vaccine, and single dose for the J&J vaccine).

There were 256 out of 5265 individuals who reported a breakthrough infection after the 2nd dose of a vaccine but prior to a booster if one was reported. Average number of days (mean ± SD) to breakthrough was 242.5 ± 123.2 days. Similarly, there were 581 out of 2,583 individuals who reported a breakthrough infection after the 1st booster dose of the vaccine but prior to a 2nd booster if one was reported.

Average number of days to breakthrough was 152.0 ± 81.7 days. Average time between first and second vaccine doses was 25.2 ± SD 18.9 days. Timing for the second dose ranged from December 16, 2020 to July 13, 2020; timing for the first booster ranged from January 21, 2021 to September 25, 2022. Demographic data for both the breakthrough and no-breakthrough groups are shown in Table 1.

**Table 1:** Demographics.

Vaccine dose	Variable	Breakthrough (N = 256)	No breakthrough (N = 5009)
Second dose	Age (median [IQR*])	47 [37–56]	53 [42–63]
	Body Mass Index, kg/m^2^ (median [IQR])	28 [24–30]	28 [24–30]
	Women (percentage)	62.90	60.60
	Weekend day vaccination (percentage)	16.40	19.00
	Moderna vaccine (percentage)	32.00	39.40
	Pfizer vaccine (percentage)	58.60	55.50
	Johnson & Johnson vaccine	9.4%	5.10%
	Average baseline TST* (median [IQR])	410.35 [472.42–445.42]	410.93 [378.00–447.10]
	Average baseline Sleep efficiency (median [IQR])	0.92 [0.89–0.93]	0.91 [0.89–0.92]
	Average baseline Awakening frequency (median [IQR])	2.94 [2.51–3.34]	2.85 [2.44–3.23]

Vaccine dose	Variable	Breakthrough (N = 581)	No breakthrough (N = 2002)
First booster	Age (median [IQR])	47 [38–58]	55 [45–65]
	Body Mass Index, kg/m^2^ (median [IQR])	28 [24–30]	27 [23–30]
	Women (percentage)	63.50	60.20
	Weekend day vaccination (percentage)	15.30	18.20
	Moderna vaccine (percentage)	45.80	47.70
	Pfizer vaccine (percentage)	53.50	51.30
	Johnson & Johnson vaccine	0.70	1.00
	Average baseline TST (median [IQR])	407.81 [374.58–441.00]	408.32 [374.83–443.17]
	Average baseline Sleep efficiency (median [IQR])	0.92 [0.89–0.93]	0.91 [0.89–0.92]
	Average baseline Awakening frequency (median [IQR])	2.99 [2.56–3.43]	2.80 [2.38–3.18]

*IQR* interquartile range, *TST* Total sleep time.

### Sleep metrics and impact on breakthrough infection

**Figure 2:**
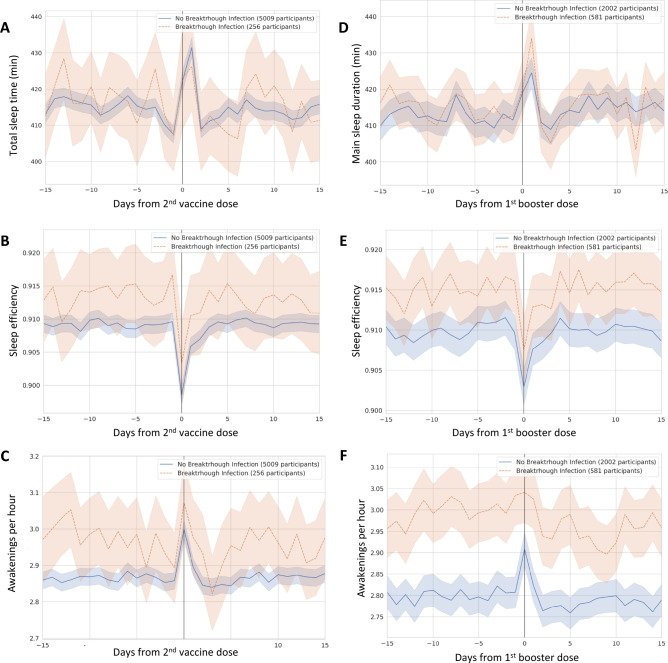
Sleep metrics in breakthrough infections. Panels (**A**)–(**C**) demonstrate temporal graphs for (**A**) Total sleep time, (**B**) Sleep efficiency, and (**C**) Awakenings per hour, for the 15 days before and after the 2nd vaccination dose (designated as day 0). Panels (**D**)–(**F**) show temporal graphs for (**D**) Total sleep time, (**E**) Sleep efficiency, and (**F**) Awakenings per hour, for the 15 days before and after the 1st booster dose (designated as day 0). Shaded areas in all graphs represent the 95% confidence interval evaluated with 10.000 bootstrap iterations.

Figure 2A–C temporally compare the sleep duration, sleep efficiency, and frequency of nighttime awakenings over the two weeks before and after the 2^nd^ vaccine dose between individuals with and without reported breakthrough infections. These sleep metrics are also temporally compared for the 1^st^ booster dose in Fig. 2D–F. We found that there were changes in sleep patterns immediately following the vaccination, as noted by the peaks and valleys in Fig. 2A–F. Table 2 compares the sleep metrics in the peri-vaccination period (defined as the three days preceding and the three days following vaccination) for both doses. We did not find a significant impact of the measured sleep metrics during the peri-vaccination period on breakthrough infection after the 2nd vaccine dose. When considering the 1st booster dose (before controlling for age, gender etc. as below), we found that an increased frequency of awakenings was associated with breakthrough infection with 3.01 ± 0.65 awakenings per hour in the breakthrough group compared to 2.82 ± 0.65 awakenings per hour in the group without reported breakthrough (*P* < 0.001 based on Mann–Whitney *U* testing). For the 1st booster, there was a statistically significant difference between groups for sleep efficiency, with 91.4% (91.1–9.17%) efficiency in the breakthrough group and 90.9% (90.7–91.0%) efficiency in the group without breakthrough infection (*P* = 0.002). Given how close these values are together, it is unclear whether this represents a clinically meaningful difference in sleep efficiency.

**Table 2:** Sleep metrics by breakthrough status for peri-vaccination period of 2^nd^ dose and 1^st^ booster.

Vaccine dose	Sleep metric	Breakthrough mean [CI]	Breakthrough (standard dev)	No breakthrough mean [CI]	No breakthrough (standard dev)	Mann–Whitney *U* P-value
Second Dose	Main sleep duration [min]	415.7 [408.3–422.86]	60	414.7 [413.1–416.4]	60.2	0.657
	Sleep efficiency	0.911 [0.907–0.916]	0.039	0.907 [0.906–0.908]	0.038	0.078
	Awakenings per hour	2.96 [2.89–3.04]	0.61	2.89 [2.87–2.90]	0.64	0.027
First Booster	Main sleep duration [min]	416.4 [411.8–421.2]	58	414.0 [411.2–416.9]	64.7	0.347
	Sleep efficiency	0.914 [0.911–0.917]	0.039	0.909 [0.907–0.911]	0.040	0.002
	Awakenings per hour	3.01 [2.96–3.06]	0.65	2.82 [2.79–2.85]	0.65	< 0.001

*CI* confidence interval.

When we examined breakthrough infections based on (1) pre-vaccination sleep, and (2) Δ sleep, we did not find a relationship for either the 2nd vaccine dose or the 1st booster (results not shown).

### Modeling impact of sleep metrics on vaccine breakthrough

**Figure 3:**
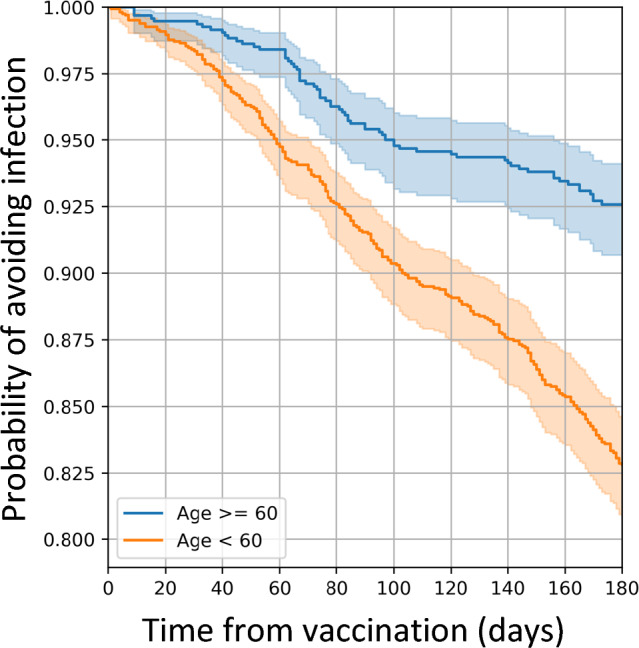
Effect of age on time to breakthrough infection. Kaplan–Meier curves constructed based on age (age < 60 years compared to age ≥ 60 years) for the first booster dose.

We performed a logistic regression analysis to further examine the predictive effects of sleep duration, sleep efficiency, and sleep awakening frequency on breakthrough infections. When we included age, gender, BMI, and weekend vs. weekday vaccination for the participants as covariates for the outcome of breakthrough infection, we found that both age and number of awakenings were consistently predictive of potential breakthrough infection after the first booster (Table 3). Finally, we examined the same variables included in the logistic regression and also included time to breakthrough from vaccination using a Cox Proportional Hazards model, which showed similar results (Table 3). Hazard ratios for breakthrough infection after first booster based on age and frequency of awakenings were 2.15 and 1.17, respectively. A Kaplan–Meier curve showing likelihood of breakthrough infection based on age is shown in Fig. 3, demonstrating that those who were younger (< 60 years) were more likely to experience breakthrough earlier compared to those who were older (≥ 60 years).

**Table 3:** Coefficients of the logistic regression models and Cox PH models predicting breakthrough status.

Vaccine dose	Equation term	Estimate	Standard Error	z value	p-value
Logistic regression modeling for second dose and 1st booster					
Second dose	(Intercept)	− 1.46	3.93 × 10^–1^	− 3.714	< 0.001
	Age	− 1.80	3.10 × 10^–1^	− 5.79	< 0.001
	Gender	− 2.91 × 10^–2^	1.38 × 10^–1^	− 2.1 × 10^–1^	0.833
	Body Mass Index	− 4.41 × 10^–5^	1.05 × 10^–2^	− 4.0 × 10^–3^	0.997
	Weekend day vaccine	− 01.74 × 10^–1^	1.73 × 10^–1^	− 1.00	0.316
	Main sleep duration	− 2.54 × 10^–2^	6.70 × 10^–2^	− 3.79 × 10^–1^	0.704
	Sleep Efficiency	5.20 × 10^–2^	5.22 × 10^–2^	9.97 × 10^–1^	0.319
	Awakenings per hour	− 3.43 × 10^–2^	1.11 × 10^–1^	− 3.10 × 10^–1^	0.756
First booster	(Intercept)	3.82 × 10^–1^	3.04 × 10^–1^	1.25	0.210
	Age	− 2.06	2.36 × 10^–1^	− 8.73	< 0.001
	Gender	− 1.31 × 10^–1^	1.04 × 10^–1^	− 1.27	0.205
	Body Mass Index	8.02 × 10^–3^	7.99 × 10^–3^	1.00	0.315
	Weekend day vaccine	− 2.39 × 10^–1^	1.32 × 10^–1^	− 1.81	0.997
	Main sleep duration	− 1.08 × 10^–3^	4.83 × 10^–2^	− 2.20 × 10^–2^	0.982
	Sleep Efficiency	6.66 × 10^–2^	3.81 × 10^–2^	1.75	0.081
	Awakenings per hour	2.20 × 10^–1^	8.15 × 10^–2^	2.70	0.007

Vaccine dose	Equation term	Hazard ratio	Standard Error	z value	p-value
Cox proportional hazards model considering time between vaccination and breakthrough					
Second dose	Age	1.97 × 10^–1^	3.01 × 10^–1^	− 5.40	< 0.001
	Gender	9.75 10^–1^	1.35 × 10^–1^	− 1.90 × 10^–1^	0.850
	Body Mass Index	9.98 × 10^–1^	1.0 × 10^–2^	− 1.98 × 10^–1^	0.843
	Weekend day vaccine	8.45 × 10^–1^	1.69 × 10^–1^	− 9.97 × 10^–1^	0.319
	Main sleep duration	0.98	6.5 × 10^–2^	2.35	0.714
	Sleep efficiency	1.07	5.06 × 10^–2^	1.42	0.155
	Awakenings per hour	9.67 × 10^–1^	1.08 × 10^–1^	− 3.14 × 10^–1^	0.753
First booster	Age	2.15 × 10^–1^	4.34 × 10^–1^	− 3.53	< 0.001
	Gender	9.04 10^–1^	8.90 × 10^–2^	− 1.14	0.255
	Body Mass Index	1.01	6.78 × 10^–3^	7.79 × 10^–1^	0.436
	Weekend day vaccine	8.15 × 10^–1^	1.15 × 10^–1^	− 1.778	0.075
	Main sleep duration	9.93 × 10^–1^	4.06 × 10^–2^	− 1.70 × 10^–1^	0.865
	Sleep efficiency	1.05	3.24 × 10^–2^	1.58	0.115
	Awakenings per hour	1.17	6.78 × 10^–2^	2.35	0.019

## Discussion

Our study uses digital biometrics to examine the impact of sleep on breakthrough infection in a real-word population. Prior studies have suggested a reduced vaccine-driven immune response with insufficient duration, though in these studies, *antibody levels, not breakthrough infections*, were measured^2,3,18,19^. Despite these limitations, these data have prompted some to call for instructions for increased sleep around the time of vaccination. Thus, our data add to the literature in important ways. First, we show that there is utility for population-level health in the passively collected sleep metrics that are measured by activity trackers. Second, contrary to our hypothesis, we did not find that sleep duration correlated with breakthrough infections. Third, our data detected a very small impact of increased awakening frequency at the time of vaccination on breakthrough after the 1st booster dose (hazard ratio of 1.17), which is of unclear significance. Fourth, our data suggest that factors other than sleep surrounding vaccination may have greater weight in determining which groups are more susceptible to breakthrough infection. For example, age appeared to predict breakthrough infection (hazard ratio of 2.15), with younger individuals (< 60 years) experiencing breakthrough earlier compared to those who were older; this could represent variations in exposures experienced by individuals in these different age categories, which could be affected by adherence to public safety measures, such as social distancing, masking, avoidance of public spaces, etc.

### Wearable data and COVID-19 vaccination

This study highlights the importance of digital health metrics in population-level health problems, such as response to vaccination, as well as the increasing importance of incorporating digital health data into clinical medicine^20,21^. Physiological and behavioral changes post-vaccination (such as heart rate, sleep, activity), with respect to the pre-vaccination baseline, are predictive of the immune response of individuals. Early proof-of-concept data from the DETECT study and from other studies confirm that consumer wearable sensors can detect the individual physiological and behavioral changes associated with the vaccination and the consequent inflammation^22,23^. In the current study, we explored sleep metrics before vaccination, which are potentially modifiable. While we did not find that the sleep metrics measured are likely to be a major driver of vaccine response, we recognize that there are limitations when measuring sleep using wearable devices. These devices do not capture detailed information on sleep staging, and do not allow for a granular evaluation of sleep data, as could be done with PSG. Regardless, our overarching hypothesis asked whether or not there was a peri-vaccination sleep-based difference between the breakthrough and on breakthrough groups, and our comparisons were made using devices with the same underlying technology. Furthermore, the metrics that we measured here are, to some degree, modifiable—for example, had a large difference between groups been found, we would have been able to suggest that individuals could potentially attempt to extend their sleep or reduce interruptions at nighttime to improve their vaccine’s effectiveness. Individuals have less control over their nightly sleep stages, or other, more detailed aspects of their sleep.

### Sleep and COVID-19 vaccination

This is one of the first studies to examine sleep and real-world breakthrough infection after COVID-19 vaccination. We did not find that sleep duration was associated with breakthrough infection in this cohort. Our findings are in line with some, but not all^18^, other human studies suggesting that inadequate sleep may not have a lasting impact on vaccine efficacy. Tufik et al. showed that severity of obstructive sleep apnea (OSA)—a form of sleep-disordered breathing that can reduce sleep duration and increase sleep fragmentation—did not appear to impact antibody levels after COVID-19 vaccination^24^. Furthermore, Benedict et al. showed that antibody titer responses to H1N1 influenza vaccination were not different after 10 days between a group that underwent sleep deprivation and a control group, although the antibody response did appear lower at earlier time points for men in the sleep deprivation group^25^. Taken together with our data, this may suggest that some aspects of sleep may play a role in the early humoral response to vaccination but that this effect may fade with time, leaving very small overall differences in longer-term vaccine efficacy. For SARS-CoV-2, antibody levels to anti-spike IgG correlate with protection from breakthrough in some, but not all, variants of the virus^26^.

While we did find a relationship between increased awakening frequency and a very slightly increased risk of breakthrough infection, the magnitude of difference between the two groups was quite small (2.8 vs. 3.0 awakenings per hour), thus making the clinical significance of these results unclear. An increased awakening frequency likely reflects decreased sleep consolidation (or more fragmented sleep), which would suggest a more unstable sleep architecture and has also been correlated with subjectively reported lower quality sleep^27^. In murine models, highly fragmented sleep has been implicated in abnormal hematopoiesis of immune progenitor cells^28^, and is associated with increased cancer cell growth^29,30^. Sleep fragmentation is also associated with an increased incidence of Alzheimer’s disease, possibly through aging of microglia, which play an important role in innate and adaptive immune responses for neurons^31,32^. Few studies exist that specifically examine the impact of fragmented sleep on immune response in vaccination, but this could be an important area of future study. It is also possible that increased awakenings per hour reflects other comorbid illnesses that we did not capture in our study.

In our study, age less than 60 years was a more important predictor of breakthrough infection compared to awakenings and that other sleep metrics in our study did not appear to impact breakthrough, which is consistent with findings published by Stærke et al. for the Delta and Omicron variants^26^. Overall, this suggests that impaired sleep may not be as important as other factors in the development of breakthrough infections. Our findings were primarily seen after the 1st booster dose, without similar results seen after the 2nd dose (although a similar trend was noted). Post-hoc power calculations suggests that this is likely due to a lower power resulting from a lower number of breakthroughs with the second dose.

### Limitations

We identify several limitations with our data set. First, data for both vaccination and breakthrough infection were self-reported. It is possible that some individuals may not have reported a breakthrough infection that occurred, and were therefore included in the “non-breakthrough”, arm of the study. However, our recently published data shows that the DETECT cohort overall had good agreement with COVID-19 infection information reported by the CDC, prior to popularity/availability of at-home testing (at which point, sensor-obtained data may have been more accurate than CDC reporting)^33^. Second, we recognize that 77% of the original cohort is excluded from analysis, although this may represent the norm for large digital medicine studies that aim to obtain sufficient objectively measured sleep/activity data; furthermore the group who completed their vaccination series may be more likely to engage with a COVID-related disease reporting app. Third, other behaviors (avoidance of public activities, masking, etc.) that impact exposure to the virus are important for breakthrough infections, but these data were not measured here. Fourth, gold-standard polysomnography was not used for sleep measurements, but we note that for basic sleep metrics, the technology utilized in activity trackers are very similar to actigraphy, which has been used previously in many sleep-related studies for general sleep measurements, including the prior vaccine work^3,11,12^. Fifth, there may be a diurnal variation in the response to vaccination^34^, which was not measured here but would be an important consideration for future studies. Sixth, the generalizability of our results may be limited by considering which demographic owns activity trackers (e.g., those who can afford and have interest in one) and engages in mobile health studies. At the same time, having a more similar group may actually reduce some of the heterogeneity inherent to this time of analysis, thus making it more likely to find a signal if one were present. Finally, the vaccines studied here were mRNA vaccines, while prior studies of hepatitis and influenza vaccines that examined the impact of sleep on antibody response were not mRNA-based. We acknowledge that we did not conduct an a priori sample size calculation due to the nature of the study, and that we may be underpowered. A much large number of events would be required to fully rule out any effect of sleep on vaccine efficacy.

Undoubtedly, sleep is important for many aspects of human health, and we did detect a small impact of awakenings from sleep on breakthrough infection. However, overall, our data suggests that sleep may not have a major impact on vaccine effectiveness for COVID-19 compared to other factors that impact breakthrough infection, such as adherence to public health measures. We would continue to recommend that shift workers or others with short sleep (on average ≤ 6 h per night) should not delay vaccination.

## Data Availability

All interested investigators will be allowed access to the analysis data set after approval of a proposal by a responsible authority at Scripps and with a data access agreement, pledging to not re-identify individuals or share the data with a third party. All data inquiries should be initially addressed to the corresponding author. Furthermore, all data generated or analysed during this study are included in the published articles cited here^22,35–37^.
